# Morrey Spaces on Domains: Different Approaches and Growth Envelopes

**DOI:** 10.1007/s12220-017-9843-y

**Published:** 2017-04-24

**Authors:** Dorothee D. Haroske, Cornelia Schneider, Leszek Skrzypczak

**Affiliations:** 1 Institute of Mathematics, Friedrich Schiller University Jena, 07737 Jena, Germany; 2 Mathematics Department, Friedrich-Alexander University Erlangen-Nüremberg, 91058 Erlangen, Germany; 3 Faculty of Mathematics and Computer Science, Adam Mickiewicz University, 61-614 Poznań, Poland

**Keywords:** Morrey spaces, Growth envelopes, Inequalities, 46E35, 47B06

## Abstract

We deal with Morrey spaces on bounded domains $\Omega$ obtained by different approaches. In particular, we consider three settings $\mathcal {M}_{u,p}(\Omega )$, $\mathbb {M}_{u,p}(\Omega )$ and $\mathfrak {M}_{u,p}(\Omega )$, where $0<p\le u<\infty$, commonly used in the literature, and study their connections and diversities. Moreover, we determine the growth envelopes $\mathfrak {E}_{\mathsf {G}}(\mathcal {M}_{u,p}(\Omega ))$ as well as $\mathfrak {E}_{\mathsf {G}}(\mathfrak {M}_{u,p}(\Omega ))$, and obtain some applications in terms of optimal embeddings. Surprisingly, it turns out that the interplay between *p* and *u* in the sense of whether $\frac{n}{u}\ge \frac{1}{p}$ or $\frac{n}{u} < \frac{1}{p}$ plays a decisive role when it comes to the behaviour of these spaces.

## Introduction

In this paper, we study Morrey spaces on bounded domains $\Omega \subset \mathbb {R}^n$ defined by different approaches. Originally, these spaces were introduced by Morrey in [21], when studying solutions of second-order quasi-linear elliptic equations in the framework of Lebesgue spaces. They can be understood as a complement (generalization) of the Lebesgue spaces $L_p(\Omega )$. In particular, the Morrey space $\mathcal {M}_{u,p}(\Omega )$, $0<p\le u<\infty$, is defined as the collection of all complex-valued Lebesgue measurable functions on $\Omega$ such that

1.1 $$\begin{aligned} \Vert f|\mathcal {M}_{u,p}(\Omega )\Vert:= & {} \sup _{x\in \Omega , j\in \mathbb {N}_0 }\left[ \mu (\Omega \cap B(x,2^{-j}))\right] ^{ \frac{1}{u}-\frac{1}{p}} \nonumber \\&\times \left[ \int _{\Omega \cap B(x,2^{-j})}|f(y)|^p\mathrm {d}y\right] ^{\frac{1}{p}}<\infty , \end{aligned}$$

cf. [14]. Obviously, $\mathcal {M}_{p,p}(\Omega )=L_p(\Omega )$, since we may assume for bounded domains that $\mathrm {diam}(\Omega )\le 1$. As can be seen from the definition, Morrey spaces investigate the local behaviour of the $L_p$ norm, which makes them useful when describing the local behaviour of solutions of non-linear partial differential equations, cf. [13, 15–20, 29]. Furthermore, applications in harmonic analysis and potential analysis can be found in the papers [2–5]. Interpolation results of these and related spaces are established in [28]. For more information we refer to the books [1] and [27].

Our aim here is to compare the spaces $\mathcal {M}_{u,p}(\Omega )$ with two other approaches for Morrey spaces on domains as can be found in the literature and characterize the unboundedness of functions belonging to the Morrey spaces $\mathcal {M}_{u,p}(\Omega )$ in some further detail.

To be more precise, we consider Morrey spaces $\mathbb {M}_{u,p}(\Omega )$, defined and studied in [23, 24], where in contrast to (1.1) the supremum is now taken over balls $B(x,2^{-j})$ fully contained in $\Omega$. We remark that in the original definition cubes were considered but the change to balls is immaterial here. On the other hand, we deal with the spaces $\mathfrak {M}_{u,p}(\Omega )$ introduced in [32], which differ from (1.1) by the fact that the supremum is now only taken over balls $B(x,2^{-j})$ having distance at least $2^{-j}$ to the boundary of $\Omega$. Clearly, by their definitions, we have embeddings $\mathcal {M}_{u,p}(\Omega )\hookrightarrow \mathbb {M}_{u,p}(\Omega )\hookrightarrow \mathfrak {M}_{u,p}(\Omega )$. Our main result in Theorem 2.7 now clarifies the connections and diversities explicitly. In particular, it turns out that

$$\begin{aligned} \mathcal {M}_{u,p}(\Omega )=\mathbb {M}_{u,p}(\Omega )=\mathfrak {M}_{u,p}(\Omega ), \quad \text {if}\quad \frac{n}{u}<\frac{1}{p}, \end{aligned}$$

and

$$\begin{aligned} \mathcal {M}_{u,p}(\Omega )\subsetneq \mathfrak {M}_{u,p}(\Omega )\quad \text {and}\quad \mathbb {M}_{u,p}(\Omega )\subsetneq \mathfrak {M}_{u,p}(\Omega ), \quad \text {if}\quad \frac{n}{u}\ge \frac{1}{p}. \end{aligned}$$

Surprisingly, we can see that the behaviour of the spaces changes with respect to the interplay of the parameters *n*, *u*, *p*.

Furthermore, in Theorem 2.3 it is established that for so-called type *A* domains, cf. Remark 2.2, the spaces $\mathcal {M}_{u,p}(\Omega )$ can be characterized by spaces $\mathcal {M}_{u,p}(\mathbb {R}^n)$ via restriction to the domain.

Apart from these considerations, we would like to understand the ‘quality’ of unboundedness, which is admitted in the spaces $\mathcal {M}_{u,p}(\Omega )$ and $\mathfrak {M}_{u,p}(\Omega )$. This contributes to the problem of optimal embeddings. We have

$$\begin{aligned} L_u(\Omega )\hookrightarrow \mathcal {M}_{u,p}(\Omega )\hookrightarrow L_p(\Omega ), \end{aligned}$$

which leads to the question whether the $L_p(\Omega )$ spaces on the right-hand side are indeed the best possible Lebesgue-type spaces in which the Morrey spaces can be embedded. These kind of questions can be investigated with the help of the *growth envelope* , where *X* is a space of functions on $\Omega$,its *growth envelope function*, and  is some additional index providing a finer description. Here $f^*$ denotes the non-increasing rearrangement of *f*. These concepts were introduced in [31] and [8], where the latter book also contains a recent survey of the present state-of-the-art (concerning extensions and more general approaches) as well as applications and further references. Therefore, our second main result can be formulated ascf. Theorem 2.13. In contrast to this we obtain for the spaces $\mathfrak {M}_{u,p}(\Omega )$ in Theorem 2.15 thatAgain, from the envelope results above it can also be seen that the interplay between the parameters in terms of $\frac{n}{u}< \frac{1}{p}$ and $\frac{n}{u}> \frac{1}{p}$ plays a decisive role in the behaviour of the Morrey spaces.

The paper is organized as follows. First we present three different approaches for Morrey spaces on domains and discuss these concepts in terms of their connections and diversities. Then we turn to the concept of growth envelopes and present and prove our main results, finally obtaining some sharp embedding results and Hardy-type inequalities.

We are very grateful to Professor Hans Triebel who introduced us in personal communications to some of his ideas contained in the unpublished notes [32]. He granted us permission to use some of his arguments and, moreover, present part of his results in the context of this paper.

## Different Approaches: Connection and Diversity

**Preliminaries** We shall adopt the following general notation: $\mathbb {N}$ denotes the set of all natural numbers, $\mathbb {N}_0=\mathbb {N}\cup \{0\}$, $\mathbb {R}^n$, $n\in \mathbb {N}$, denotes the *n*-dimensional real Euclidean space. Furthermore, $\mu =|\cdot |$ stands for the Lebesgue measure. For a real number *a*, let $a_+:=\max (a,0)$ and let $\lfloor a \rfloor$ denote its integer part. For $p\in (0,\infty ]$, the number $p'$ is defined by $1/p':=(1-1/p)_+$ with the convention that $1/\infty =0$. By *c*, $c_1$, $c_2$, etc. we denote positive constants independent of appropriate quantities. For two non-negative expressions (i.e. functions or functionals) ${\mathcal {A}}$, ${\mathcal {B}}$, the symbol ${\mathcal {A}}\lesssim {\mathcal {B}}$ (or ${\mathcal {A}}\gtrsim {\mathcal {B}}$) means that ${\mathcal {A}}\le c\, {\mathcal {B}}$ (or $c\,{\mathcal {A}}\ge {\mathcal {B}}$). If ${\mathcal {A}}\lesssim {\mathcal {B}}$ and ${\mathcal {A}}\gtrsim {\mathcal {B}}$, we write ${\mathcal {A}}\sim {\mathcal {B}}$ and say that ${\mathcal {A}}$ and ${\mathcal {B}}$ are equivalent. Given two quasi-Banach spaces *X* and *Y*, we write $X\hookrightarrow Y$ if $X\subset Y$ and the natural embedding is bounded.

**Different Approaches** In this section we discuss three different approaches for Morrey spaces on domains. They provide intrinsic and extrinsic characterizations and we show below that under some restrictions on the parameters involved, the introduced spaces may coincide or differ.

We assume throughout this paper that the domain $\Omega \subset \mathbb {R}^n$ is bounded.

Let

2.1 $$\begin{aligned} S_J:=\{ x\in \Omega : \ 2^{-J+1}<\mathrm {dist}(x,\partial \Omega )<2^{-J+3}\}, \end{aligned}$$

$J\in \mathbb {N}_0$, where we may assume that

$$\begin{aligned} \Omega =\bigcup _{J=0}^{\infty }S_J, \qquad S_0\ne \emptyset . \end{aligned}$$

If $\Omega$ is a Lipschitz domain, then $|S_J|\sim 2^{-J}$.

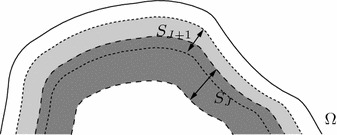



Let $M(\Omega )$ be the collection of all equivalence classes of complex-valued Lebesgue measurable functions on $\Omega$. There are several equivalent definitions of Morrey spaces. One can take averages over balls or cubes, or dyadic cubes. Below we give the definition of the spaces using balls $B(x,2^{-j})$ centred at $x\in \Omega$ and of radius $2^{-j}$, $j\in \mathbb {N}_0$, but in some proofs we use also the equivalent norm that uses dyadic cubes.

### Definition 2.1

Let $\Omega \subset \mathbb {R}^n$ be a bounded domain and $0<p\le u<\infty$.

(i) The Morrey space $\mathcal {M}_{u,p}(\Omega )$ is defined to be the set of all functions $f\in M(\Omega )$ such that
  2.2 $$\begin{aligned} \Vert f|\mathcal {M}_{u,p}(\Omega )\Vert:= & {} \sup _{x\in \Omega , j\in \mathbb {N}_0 }\left[ \mu (\Omega \cap B(x,2^{-j}))\right] ^{ \frac{1}{u}-\frac{1}{p} } \nonumber \\&\times \left[ \int _{\Omega \cap B(x,2^{-j})}|f(y)|^p\mathrm {d}y\right] ^{\frac{1}{p}}<\infty . \end{aligned}$$
(ii) The Morrey space $\mathbb {M}_{u,p}(\Omega )$ is defined to be the set of all functions $f\in M(\Omega )$ such that
  2.3 $$\begin{aligned} \Vert f|\mathbb {M}_{u,p}(\Omega )\Vert =\sup _{x\in \Omega , B(x,2^{-j})\subset \Omega }2^{-jn\left( \frac{1}{u}-\frac{1}{p}\right) }\left[ \int _{B(x,2^{-j})}|f(y)|^p\mathrm {d}y\right] ^{\frac{1}{p}}<\infty . \end{aligned}$$
(iii) The Morrey space $\mathfrak {M}_{u,p}(\Omega )$ is defined to be the set of all functions $f\in M(\Omega )$ such that
  2.4 $$\begin{aligned} \Vert f|\mathfrak {M}_{u,p}(\Omega )\Vert =\sup _{x\in \Omega , j\ge j_x}2^{-jn\left( \frac{1}{u}-\frac{1}{p}\right) }\left[ \int _{B(x,2^{-j})}|f(y)|^p\mathrm {d}y\right] ^{\frac{1}{p}}<\infty , \end{aligned}$$
  where for $x\in \Omega$, by $j_x$ we denote the smallest number such that
  2.5 $$\begin{aligned} \mathrm {dist}\left( B(x,2^{-j}),\partial \Omega \right) \ge 2^{-j}\quad \text {if}\quad j_x\le j\in \mathbb {N}. \end{aligned}$$

### Remark 2.2

(i) The spaces $\mathcal {M}_{u,p}(\Omega )$ are adapted from [14, Definition 4.3.3]. It is clear from the definition that they can be considered as a complement to $L_p$ spaces. Clearly, we have $\mathcal {M}_{p,p}(\Omega )=L_p(\Omega )$ with $p\in (0,\infty )$. The definition of the spaces $\mathbb {M}_{u,p}(\Omega )$ was already considered in [23], and the last approach for $\mathfrak {M}_{u,p}(\Omega )$ was considered in [32], where also growth envelopes for these spaces were studied. Our approach differs from the above ones in the sense that we consider parameters $0<p\le u<\infty$, which is more convenient for us, whereas the above references deal with $0<p<\infty$ and parameters $\lambda :=-n p\left( \frac{1}{u}-\frac{1}{p}\right)$ or $\sigma =-\frac{n}{u}$, resulting in the conditions $0\le \lambda \le n$ or $-\frac{n}{p}\le \sigma \le 0$, respectively.
(ii) Clearly we have the embeddings
  2.6 $$\begin{aligned} \mathcal {M}_{u,p}(\Omega )\hookrightarrow \mathbb {M}_{u,p}(\Omega )\hookrightarrow \mathfrak {M}_{u,p}(\Omega ), \end{aligned}$$
  which follows directly from the definitions of the spaces. Obviously, $\mathcal {M}_{u,p}(\mathbb {R}^n)={\mathbb {M}}_{u,p}(\mathbb {R}^n)$.
(iii) In order to be able to compare the Morrey spaces $\mathcal {M}_{u,p}(\Omega )$ as defined in (i) with the other two Morrey spaces on domains, we shall restrict ourselves to so-called domains *of type A* meaning that there exists a constant $A>0$ such that for every $x\in \overline{\Omega }$ and all $j\ge j_0$ we have
  $$\begin{aligned} \mu (\Omega \cap B(x,2^{-j}))\ge A2^{-jn}. \end{aligned}$$
  This approach already appears in [33, Ch. 1] for the definition of Morrey spaces (when $p=2$). In this case (2.2) reduces to
  2.7 $$\begin{aligned} \Vert f|\mathcal {M}_{u,p}(\Omega )\Vert \sim \sup _{x\in \Omega , j\in \mathbb {N}_0 }2^{-jn\left( \frac{1}{u}-\frac{1}{p}\right) }\left[ \int _{\Omega \cap B(x,2^{-j})}|f(y)|^p\mathrm {d}y\right] ^{\frac{1}{p}}<\infty . \end{aligned}$$
  For example a square in the plane is a set of type *A* with $A=\frac{1}{2}$, whereas the domain $\Omega =\{(x,y)\in \mathbb {R}^2: \ 0<x<1, \ 0<y<x^2\}$ is not of type *A* for any $A>0$ (since the origin is a cuspidal point of the boundary of $\Omega$). The situations are illustrated below. 
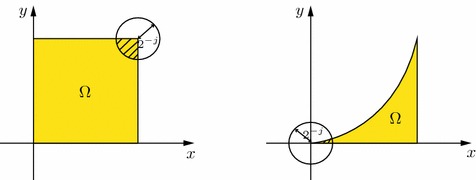
Furthermore, our definitions in (i), (ii) differ from the ones used in [14, 23] in the sense that we take balls with radii $2^{-j}$, $j\ge j_0$ instead of $r\in (0,\delta )$. Furthermore, we take the supremum over all $j\in \mathbb {N}_0$ instead of $j\ge j_0$ only, since for functions $f\in L_p(\Omega )$ we clearly have that the term with $j=0$ is finite and for $0\le j\le j_0$ we have
  $$\begin{aligned} 2^{-jn\left( \frac{1}{u}-\frac{1}{p}\right) }\left[ \int _{\Omega \cap B(x,2^{-j})}|f(y)|^p\mathrm {d}y\right] ^{\frac{1}{p}} \le 2^{-j_0n\left( \frac{1}{u}-\frac{1}{p}\right) }\left[ \int _{\Omega \cap B(x,1)}|f(y)|^p\mathrm {d}y\right] ^{\frac{1}{p}}, \end{aligned}$$
  which differs from the $j=0$ term only by some constant depending on $j_0$.

We proceed by demonstrating that the spaces $\mathcal {M}_{u,p}(\Omega )$ can be characterized by spaces $\mathcal {M}_{u,p}(\mathbb {R}^n)$ normed by

$$\begin{aligned} \Vert g|\mathcal {M}_{u,p}(\mathbb {R}^n)\Vert =\sup _{x\in \mathbb {R}^n, j\in \mathbb {Z}}2^{-jn\left( \frac{1}{u}-\frac{1}{p}\right) }\left[ \int _{B(x,2^{-j})}|g(y)|^p\mathrm {d}y\right] ^{\frac{1}{p}}, \end{aligned}$$

via restriction to the domain.

### Theorem 2.3

Let $\Omega \subset \mathbb {R}^n$ be a type *A* domain and $0<p\le u<\infty$. Let $f\in \mathcal {M}_{u,p}(\Omega )$, then

$$\begin{aligned} \Vert f|\mathcal {M}_{u,p}(\Omega )\Vert \sim \inf \Vert g|\mathcal {M}_{u,p}(\mathbb {R}^n)\Vert , \end{aligned}$$

where the infimum is taken over all $g\in \mathcal {M}_{u,p}(\mathbb {R}^n)$ such that $g\big |_{\Omega }=f$.

### Proof

Consider

$$\begin{aligned} \tilde{f}= {\left\{ \begin{array}{ll} f,&{} x\in \Omega ,\\ 0,&{} x\in \mathbb {R}^n\setminus \Omega . \end{array}\right. } \end{aligned}$$

Clearly $\Vert f|\mathcal {M}_{u,p}(\Omega )\Vert \le \Vert \tilde{f}|\mathcal {M}_{u,p}(\mathbb {R}^n)\Vert$ so we are left to prove the converse. By definition

$$\begin{aligned} \Vert \tilde{f}|\mathcal {M}_{u,p}(\mathbb {R}^n)\Vert =\sup _{x\in \mathbb {R}^n, j\in \mathbb {Z}}2^{-jn\left( \frac{1}{u}-\frac{1}{p}\right) }\left[ \int _{B(x,2^{-j})\cap \Omega }|f(y)|^p\mathrm {d}y\right] ^{\frac{1}{p}}. \end{aligned}$$

First we argue why it is always sufficient to consider $x\in \Omega$ instead of $x\in \mathbb {R}^n$. Let $x\notin \Omega$.

- If $\mathrm {dist}(x,\partial \Omega )>2^{-j}$, then our balls lie outside of $\Omega$, i.e. $\Omega \cap B(x,2^{-j})=\emptyset$ and our integral reduces to zero.
- If $\mathrm {dist}(x,\partial \Omega )<2^{-j}$, our balls intersect with $\Omega$. But in this case it is always possible to choose $y\in \Omega$ such that $B(x,2^{-j})\subset B(y,2^{-j+1})$ and calculate
  $$\begin{aligned} \Vert \tilde{f}|\mathcal {M}_{u,p}(\mathbb {R}^n)\Vert= & {} \sup _{x\in \mathbb {R}^n, j\in \mathbb {Z}}2^{-jn\left( \frac{1}{u}-\frac{1}{p}\right) }\left[ \int _{B(x,2^{-j})\cap \Omega }|f(y)|^p\mathrm {d}y\right] ^{\frac{1}{p}}\\\le & {} \sup _{y\in \Omega , j\in \mathbb {Z}}2^{-n\left( \frac{1}{u}-\frac{1}{p}\right) }2^{(-j+1)n\left( \frac{1}{u}-\frac{1}{p}\right) }\\&\times \,\left[ \int _{B(y,2^{-j+1})\cap \Omega }|f(y)|^p\mathrm {d}y\right] ^{\frac{1}{p}}\\= & {} C_{n,u,p}\sup _{y\in \Omega , j\in \mathbb {Z}}2^{(-j+1)n\left( \frac{1}{u}-\frac{1}{p}\right) }\left[ \int _{B(y,2^{-j+1})\cap \Omega }|f(y)|^p\mathrm {d}y\right] ^{\frac{1}{p}}. \end{aligned}$$

It remains to show that the supremum is attained for some $j\in \mathbb {N}_0$. Since $\Omega$ is bounded, w.l.o.g. we can assume that it can be covered by some ball with radius 1. Then for big radii corresponding to $j<0$ there is some $x\in \Omega$ such that $\Omega \subset B(x,2^{-j})$. Thus we see that

$$\begin{aligned} \sup _{x\in \Omega , -j\in \mathbb {N}}2^{-jn\left( \frac{1}{u}-\frac{1}{p}\right) }\left[ \int _{B(x,2^{-j})\cap \Omega }|f(y)|^p\mathrm {d}y\right] ^{\frac{1}{p}}\le 1\cdot \left[ \int _{\Omega }|f(y)|^p\mathrm {d}y\right] ^{\frac{1}{p}}, \end{aligned}$$

which corresponds to some term which can be expressed by level $j=0$. Therefore, we have shown that

$$\begin{aligned} \Vert \tilde{f}|\mathcal {M}_{u,p}(\mathbb {R}^n)\Vert= & {} \sup _{x\in \mathbb {R}^n, j\in \mathbb {Z}}2^{-jn\left( \frac{1}{u}-\frac{1}{p}\right) }\left[ \int _{B(x,2^{-j})\cap \Omega }|f(y)|^p\mathrm {d}y\right] ^{\frac{1}{p}}\\\le & {} \sup _{x\in \Omega , j\in \mathbb {N}_0}2^{-jn\left( \frac{1}{u}-\frac{1}{p}\right) }\left[ \int _{B(x,2^{-j})\cap \Omega }|f(y)|^p\mathrm {d}y\right] ^{\frac{1}{p}} \\= & {} \Vert f|\mathcal {M}_{u,p}(\Omega )\Vert , \end{aligned}$$

where we have finally used the assumption on $\Omega$ to be a domain of type *A*. This completes the proof. $\square$

Next we briefly report on a result of Piccinini in [23], see also [24], for spaces $\mathbb {M}_{u,p}(\Omega )$. We adapt the formulation to our setting and extend it to the quasi-Banach case which causes no difficulties looking at the proof. Let $Q\subset \mathbb {R}^n$ be some cube, and $0<p_i\le u_i<\infty$, $i=1,2$. Then

2.8 $$\begin{aligned} {\mathbb {M}}_{u_1,p_1}(Q) \hookrightarrow {\mathbb {M}}_{u_2,p_2}(Q)\qquad \text {if, and only if,}\qquad p_2\le p_1 \quad \text {and}\quad u_2\le u_1. \end{aligned}$$

The result (2.8) was extended to $\mathbb {R}^n$ by Rosenthal in [26, Satz 1.6],

2.9 $$\begin{aligned} {\mathcal {M}}_{u_1,p_1}(\mathbb {R}^n) \hookrightarrow {\mathcal {M}}_{u_2,p_2}(\mathbb {R}^n) \qquad \text {if, and only if,}\qquad p_2\le p_1\le u_1=u_2, \end{aligned}$$

recall $$\mathcal {M}_{u,p}(\mathbb {R}^n)=\mathbb {M}_{u,p}(\mathbb {R}^n)$$.

### Remark 2.4

Note that in [24] also Morrey spaces of type $\mathcal {M}_{u,p}(\Omega )$ for domains of type *A* are studied, whereas in [23] the setting is restricted to cubes only which simplifies the situation. Furthermore, in [24] one can find further generalizations of this approach, as well as related interpolation results.

**Some Properties of the Spaces** $\mathfrak {M}_{u,p}(\Omega )$ We collect some properties of the spaces $\mathfrak {M}_{u,p}(\Omega )$ that can be found in the unpublished notes [32, Sect. 2.3]. By standard arguments it follows that (2.4) are quasi-Banach spaces. The restriction of the parameters in terms of $0<p\le u<\infty$ makes sense. In particular, extending the definition of the spaces to $u=\infty$, by a Lebesgue point argument we have that

$$\begin{aligned} \mathfrak {M}_{\infty , p}(\Omega )=L_{\infty }(\Omega ), \end{aligned}$$

whereas for $u<p$ the corresponding norm becomes

$$\begin{aligned} \Vert f|\mathfrak {M}_{u,p}(\Omega )\Vert =\sup _{J\in \mathbb {N}_0, x\in S_J}2^{-Jn\left( \frac{1}{u}-\frac{1}{p}\right) }\left[ \int _{B(x,2^{-J})}|f(y)|\mathrm {d}y\right] ^{\frac{1}{p}}, \end{aligned}$$

but there is no longer additional local information as in (2.4). The following theorem collects some embedding assertions obtained by Triebel [32].

### Theorem 2.5

Let $\Omega \subset \mathbb {R}^n$ be a bounded domain.

(i) Let $$0<p_i\le u_i<\infty $$, $$i=1,2$$. Then
  2.10 $$\begin{aligned} \mathfrak {M}_{u_1,p_1}(\Omega )\hookrightarrow \mathfrak {M}_{u_2,p_2}(\Omega ), \quad \text {if}\quad p_2\le p_1, \ u_2\le u_1. \end{aligned}$$
(ii) If, in addition, $\Omega$ is a bounded Lipschitz domain, then
  2.11 $$\begin{aligned} \mathfrak {M}_{u,p}(\Omega )\hookrightarrow L_{\frac{u}{n},\infty }(\Omega ), \qquad \text {if }\quad \frac{n}{u}>\frac{1}{p}. \end{aligned}$$

### Proof

(i) The embedding follows from the definition of the spaces and Hölder’s inequality. To be more precise, $p_1\ge p_2$ implies that
  $$\begin{aligned} \Vert f|\mathfrak {M}_{u_2,p_2}(\Omega )\Vert= & {} \sup _{x\in \Omega , j\ge j_x}2^{-jn\left( \frac{1}{u_2}-\frac{1}{p_2}\right) }\left( \int _{ B(x,2^{-j})}|f(y)|^{p_2}\mathrm {d}y\right) ^{\frac{1}{p_2}}\\\le & {} \sup _{x\in \Omega , j\ge j_x}2^{-jn\left( \frac{1}{u_2}-\frac{1}{p_2}\right) }\left( \int _{ B(x,2^{-j})}|f(y)|^{p_1}\mathrm {d}y\right) ^{\frac{1}{p_1}} \\&\times \left( 2^{-jn}\right) ^{\frac{1-\frac{p_2}{p_1}}{p_2}}\\= & {} \sup _{x\in \Omega , j\ge j_x}2^{-jn\left( \frac{1}{u_1}-\frac{1}{p_1}\right) } \underbrace{2^{-jn\left( \frac{1}{u_2}-\frac{1}{u_1}-\frac{1}{p_2}+\frac{1}{p_1}+\frac{1}{p_2}-\frac{1}{p_1}\right) }}_{\le 1} \\&\times \left( \int _{ B(x,2^{-j})}|f(y)|^{p_1}\mathrm {d}y\right) ^{\frac{1}{p_1}}\\\le & {} \Vert f|\mathfrak {M}_{u_1,p_1}(\Omega )\Vert . \end{aligned}$$
(ii) The proof can be found in [32, Th. 2.15] and uses arguments from interpolation theory. We sketch the main ideas. Let
  2.12 $$\begin{aligned} d(x)=\mathrm {dist}(x,\partial \Omega ), \qquad x\in \Omega \end{aligned}$$
  and $S_J$ as in (2.1) with $|S_J|\sim 2^{-J}$. Then
  2.13 $$\begin{aligned} d^{-\varkappa }\in L_{\frac{1}{\varkappa },\infty }(\Omega ), \qquad \varkappa >0, \end{aligned}$$
  where $L_{\frac{1}{\varkappa },\infty }(\Omega )$ denotes a Lorentz space. Recall that
  2.14 $$\begin{aligned} L_{\frac{1}{\varkappa },\infty }(\Omega )\cdot L_p(\Omega )\hookrightarrow L_{r,\infty }(\Omega ), \qquad 0<\frac{1}{r}=\frac{1}{p}+\varkappa . \end{aligned}$$
  This is well-known, a short detailed proof of this assertion can also be found in [7, Lem. 2.12] and is based on Hölder’s inequality and real interpolation of Lebesgue and Lorentz spaces. Then it follows from (2.13) that
  2.15 $$\begin{aligned} \Vert f|L_{r,\infty }(\Omega )\Vert \le c\Vert d^{\varkappa }f|L_p(\Omega )\Vert , \qquad 0<\frac{1}{r}=\frac{1}{p}+\varkappa . \end{aligned}$$
  Using again the fact that $\Omega$ is a bounded Lipschitz domain one obtains
  2.16 $$\begin{aligned} \int _{\Omega }d^{\varkappa p}(x)|f(x)|^p\mathrm {d}x\le & {} c\sum _{J=0}^{\infty }2^{-\varkappa pJ+J(n-1)}\sup _{x\in S_J}\int _{B(x,2^{-J})}|f(y)|^p\mathrm {d}y \nonumber \\\le & {} c\sum _{J=0}^{\infty }2^{Jn-J\frac{n}{u}p}\sup _{x\in S_J}\int _{B(x,2^{-J})}|f(y)|^p\mathrm {d}y \end{aligned}$$
  with
  $$\begin{aligned} -\frac{n}{u}=-\varkappa -\frac{1}{p} \end{aligned}$$
  which implies $r=\frac{u}{n}$ in (2.14). If $\frac{n}{u}>\frac{1}{p}$, then $\varkappa =\frac{n}{u}-\frac{1}{p}>0$ as requested in (2.12). Combining (2.15) and (2.16) we have
  2.17 $$\begin{aligned} \Vert f|L_{\frac{u}{n},\infty }(\Omega )\Vert \le c\left( \sum _{J=0}^{\infty } 2^{-J\frac{n}{u}p} \left( \sup _{x\in S_J}2^{Jn}\int _{B(x,2^{-J})}|f(y)|^p\mathrm {d}y\right) \right) ^{1/p}. \end{aligned}$$
  Let $\mathbb {L}_{u,p}(\Omega )$ be a space quasi-normed by the right-hand side of the last inequality. Thus (2.17) means that
  2.18 $$\begin{aligned} \mathbb {L}_{u,p}(\Omega ) \hookrightarrow L_{\frac{u}{n},\infty }(\Omega )\ . \end{aligned}$$
  We take now (2.18) as a starting point for real interpolation. The interpolation of spaces $\mathbb {L}_{u,p}(\Omega )$ can be described in the same way as the interpolation of weighted sequence spaces so we recall it briefly. Let *A* be a quasi-Banach space, $0<q\le \infty$ and $\delta \in \mathbb {R}$. Then $\ell ^{\delta }_q(A)$ is the quasi-Banach space consisting of all sequences $\xi =\left\{ \xi _j\right\} _{j=0}^{\infty }\subset A$ such that
  $$\begin{aligned} \Vert \xi |\ell ^{\delta }_q(A)\Vert =\left( \sum _{j=0}^{\infty }2^{j\delta q}\Vert \xi _j|A\Vert ^q\right) ^{1/q}<\infty \end{aligned}$$
  (with obvious modifications if $q=\infty$). Let
  $$\begin{aligned} q_0,q_1,q\in (0,\infty ], \qquad -\infty<\delta _0<\delta _1<\infty \quad \text {and}\quad 0<\theta <1. \end{aligned}$$
  Then in [22], cf. also [30, Sect. 1.18.2] and [6, Th. 5.6.1], it is shown that
  $$\begin{aligned} \left( \ell ^{\delta _0}_{q_0}(A), \ell ^{\delta _1}_{q_1}(A)\right) _{\theta ,q}=\ell ^{\delta }_q(A), \qquad \delta =(1-\theta )\delta _0+\theta \delta _1. \end{aligned}$$
  Adopting the proof of Theorem 5.6.1 in [6] to our situation we get
  $$\begin{aligned} \left( \mathbb {L}_{u_0,p}(\Omega ) ,\mathbb {L}_{u_1, p} (\Omega ) \right) _{\theta , \infty } = \mathfrak {M}_{u , p}(\Omega ) , \qquad \frac{n}{u}=(1-\theta )\frac{n}{u_0}+\theta \frac{n}{u_1} . \end{aligned}$$
  Using the well-known interpolation properties of Lorentz spaces
  $$\begin{aligned} \left( L_{\frac{u_0}{n},\infty }(\Omega ),L_{\frac{u_1}{n},\infty }(\Omega )\right) _{\theta , \infty }=L_{\frac{u}{n},\infty }(\Omega ), \qquad \frac{n}{u}=(1-\theta )\frac{n}{u_0}+\theta \frac{n}{u_1}, \end{aligned}$$
  we finally obtain the desired result
  $$\begin{aligned} \Vert f|L_{\frac{u}{n},\infty }(\Omega )\Vert\le & {} c \sup _{J\in \mathbb {N}_0,x\in S_J}2^{-Jn\left( \frac{1}{u}-\frac{1}{p}\right) }\left( \int _{B(x,2^{-J})}|f(y)|^p\mathrm {d}y\right) ^{1/p} \\= & {} c\Vert f|\mathfrak {M}_{u,p}(\Omega )\Vert . \end{aligned}$$

$$\square $$

### Remark 2.6

In [32] some further Morrey spaces on domains were introduced, $\mathfrak {M}^*_{u,p}(\Omega )$, as the set of all functions $f\in M(\Omega )$ such that

2.19 $$\begin{aligned} \Vert f|\mathfrak {M}^*_{u,p}(\Omega )\Vert = \left[ \sum _{J=0}^\infty \ \sup _{x\in S_J, j\ge J}2^{-jnp\left( \frac{1}{u}-\frac{1}{p}\right) }\int _{B(x,2^{-j})}|f(y)|^p\mathrm {d}y\right] ^{\frac{1}{p}}<\infty . \end{aligned}$$

They are obviously contained in $\mathfrak {M}_{u,p}(\Omega )$, since the $\ell _\infty$-norm in (2.4) is replaced now by its $\ell _p$ counterpart in (2.19). Part (i) of Theorem 2.5 is literally the same for the spaces $\mathfrak {M}^*_{u,p}(\Omega )$, whereas part (ii), that is, (2.11), has to be replaced by

2.20 $$\begin{aligned} \mathfrak {M}^*_{u,p}(\Omega )\hookrightarrow L_{\frac{u}{n},p}(\Omega ), \qquad \text {if }\quad \frac{n}{u}\ge \frac{1}{p}, \end{aligned}$$

where $\Omega$ is a bounded Lipschitz domain, $0<p\le u<\infty$. In particular,

$$\begin{aligned} \mathfrak {M}^*_{np,p}(\Omega ) \hookrightarrow L_p(\Omega ),\quad 0<p<\infty . \end{aligned}$$

**Connection and Diversity** Now we take a closer look at the connections and diversities of these spaces refining the embedding result (2.6). Surprisingly, it turns out that depending on the parameters *n*, *u*, and *p* the three approaches might coincide altogether or differ completely. The precise results can be found below.

### Theorem 2.7

Let $\Omega \subset \mathbb {R}^n$ be a bounded Lipschitz domain, and $0<p\le u<\infty$.

- (i)    If $$\frac{n}{u}<\frac{1}{p}$$, then
  $$\begin{aligned} \mathcal {M}_{u,p}(\Omega )=\mathbb {M}_{u,p}(\Omega )=\mathfrak {M}_{u,p}(\Omega ). \end{aligned}$$
- (ii)    If $\frac{n}{u}\ge \frac{1}{p}$, then
  $$\begin{aligned} \mathbb {M}_{u,p}(\Omega )\subsetneq \mathfrak {M}_{u,p}(\Omega ), \end{aligned}$$
  in particular, also
  $$\begin{aligned} \mathcal {M}_{u,p}(\Omega )\subsetneq \mathfrak {M}_{u,p}(\Omega ). \end{aligned}$$

### Proof

Note that our assumption of $\Omega$ to be a bounded Lipschitz domain implies that $\Omega$ is also a type *A* domain, cf. [33, Ch. 1, p.32].

*Step 1.*   We first show (i). By (2.6) it suffices to show that for any $f\in \mathfrak {M}_{u,p}(\Omega )$ we have

$$\begin{aligned} \Vert f|\mathbb {M}_{u,p}(\Omega )\Vert \lesssim \Vert f|\mathfrak {M}_{u,p}(\Omega )\Vert . \end{aligned}$$

Having a closer look at the norms of the two spaces we need to show that balls $B(x,2^{-j})\subset \Omega$, which can be arbitrarily close to the boundary $\partial \Omega$ and are considered in the supremum of $\mathbb {M}_{u,p}(\Omega )$, can be ‘compensated’ somehow with the help of balls $B(x,2^{-j})$ with $j\ge j_x$ as allowed in the supremum of $\mathfrak {M}_{u,p}(\Omega )$.

This can be seen as follows.

Consider the sets $S_J$ from (2.1). We cover balls $B(x,2^{-j})\subset \Omega$ on their intersection with $S_J$ by balls $B(\tilde{x},2^{-J})$ with $\tilde{x}\in S_J$, $J\ge J_{\tilde{x}}\ge j$, and control the number of balls of radius ${2^{-J}}$ we need. Since the domain is bounded and Lipschitz the volume of the intersection is at most $2^{-J}2^{-j(n-1)}$. So the intersection can be covered by $C 2^{J(n-1)}2^{-j(n-1)}$ balls of radius $2^{-J}$, where the constant *C* is independent of *j* and *J*. 
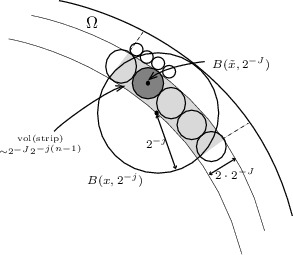


This leads to the estimate

2.21 $$\begin{aligned} \left( \int _{B(x,2^{-j})}|f(y)|^p\mathrm {d}y\right) ^{\frac{1}{p}}\lesssim & {} \left( \sum _{J=j}^{\infty }2^{J(n-1)}2^{-j(n-1)} \sup _{\tilde{x}\in S_J} \int _{B(\tilde{x},2^{-J})}|f(y)|^p\mathrm {d}y\right) ^{\frac{1}{p}} \nonumber \\\le & {} \left( \sum _{J=j}^{\infty }2^{J(n-1)}2^{-j(n-1)}2^{Jn\left( \frac{1}{u}-\frac{1}{p}\right) p}\Vert f|\mathfrak {M}_{u,p}(\Omega )\Vert ^p\right) ^{\frac{1}{p}} \nonumber \\= & {} 2^{-j\frac{(n-1)}{p}}\left( \sum _{J=j}^{\infty }2^{-J\left( 1-\frac{np}{u}\right) }\right) ^{\frac{1}{p}}\Vert f|\mathfrak {M}_{u,p}(\Omega )\Vert \nonumber \\\sim & {} 2^{-j\frac{(n-1)}{p}}2^{-j\left( \frac{1}{p}-\frac{n}{u}\right) }\Vert f|\mathfrak {M}_{u,p}(\Omega )\Vert \nonumber \\= & {} 2^{jn\left( \frac{1}{u}-\frac{1}{p}\right) }\Vert f|\mathfrak {M}_{u,p}(\Omega )\Vert , \end{aligned}$$

where in the second but last step we used that the exponent of our geometric series is negative since $\frac{n}{u}<\frac{1}{p}$. Bringing the weight factor in (2.21) to the left-hand side we obtain the desired result,

$$\begin{aligned} \Vert f|\mathbb {M}_{u,p}(\Omega )\Vert= & {} \sup _{x\in \Omega , B(x,2^{-j})\subset \Omega }2^{-jn\left( \frac{1}{u}-\frac{1}{p}\right) }\left( \int _{B(x,2^{-j})}|f(y)|^p\mathrm {d}y\right) ^{\frac{1}{p}} \\\lesssim & {} \Vert f|\mathfrak {M}_{u,p}(\Omega )\Vert . \end{aligned}$$

To show the coincidence $\mathcal {M}_{u,p}(\Omega )=\mathbb {M}_{u,p}(\Omega )$ in (i) we may stress the same arguments as above, the only difference being (in the picture) that now we cover a ball centred at $x\in \partial \Omega$ with balls $B(\tilde{x},2^{-J})$, where $J\ge J_x$. The calculations remain the same.

*Step 2.*   As for (ii) it will be enough to show that we can find a function $f\in \mathfrak {M}_{u,p}(\Omega )$ with $f\notin \mathbb {M}_{u,p}(\Omega )$. Consider

$$\begin{aligned} f(x)=d(x)^{-\frac{n}{u}},\qquad \text {where } d(x)=\mathrm {dist}(x,\partial \Omega ). \end{aligned}$$

Then $$f\in \mathfrak {M}_{u,p}(\Omega )$$, since

$$\begin{aligned} \Vert f|\mathfrak {M}_{u,p}(\Omega )\Vert= & {} \sup _{x\in \Omega , j\ge j_x}2^{-jn\left( \frac{1}{u}-\frac{1}{p}\right) }\left[ \int _{\Omega \cap B(x,2^{-j})}d(y)^{-\frac{np}{u}}\mathrm {d}y\right] ^{\frac{1}{p}}\\\le & {} \sup _{x\in \Omega , j\ge j_x}2^{-jn\left( \frac{1}{u}-\frac{1}{p}\right) }\left[ (2^{-j})^{-\frac{np}{u}}\cdot 2^{-jn}\right] ^{\frac{1}{p}}\le 1, \end{aligned}$$

where in the second step we used as estimate the largest value of *d*(*y*) in the ball $B(x,2^{-j})$ with $j\ge j_x$. On the other hand we have $f\notin \mathbb {M}_{u,p}(\Omega )$ which can be seen as follows. Consider the disjoint sets

$$\begin{aligned} \tilde{S}_J=\{x\in \Omega : 2^{-J+1}< \mathrm {dist}(x,\partial \Omega )\le 2^{-J+2}\}, \qquad J\in \mathbb {N}_0, \end{aligned}$$

where we assume that

$$\begin{aligned} \Omega =\bigcup _{J=0}^{\infty }S_J, \qquad S_0\ne \emptyset . \end{aligned}$$

Then we calculate

$$\begin{aligned} \Vert f|\mathbb {M}_{u,p}(\Omega )\Vert= & {} \sup _{x\in \Omega , B(x,2^{-j})\subset \Omega }2^{-jn\left( \frac{1}{u}-\frac{1}{p}\right) }\left[ \int _{B(x,2^{-j})}d(y)^{-\frac{np}{u}}\mathrm {d}y\right] ^{\frac{1}{p}}\\\ge & {} \sup _{x\in \Omega , B(x,2^{-j})\subset \Omega }2^{-jn\left( \frac{1}{u}-\frac{1}{p}\right) }\left[ \sum _{J=j}^{\infty }\int _{B(x,2^{-j})\cap \tilde{S}_J}d(y)^{-\frac{np}{u}}\mathrm {d}y\right] ^{\frac{1}{p}}\\\sim & {} \sup _{x\in \Omega , B(x,2^{-j})\subset \Omega }2^{-jn\left( \frac{1}{u}-\frac{1}{p}\right) }\left[ \sum _{J=j}^{\infty }2^{J\frac{np}{u}}2^{-J}2^{-j(n-1)}\right] ^{\frac{1}{p}}\\= & {} \sup _{j\in \mathbb {N}_0}2^{-j\left( \frac{n}{u}-\frac{1}{p}\right) }\left[ \sum _{J=j}^{\infty }2^{J\left( \frac{np}{u}-1 \right) }\right] ^{\frac{1}{p}}=\infty ,\\ \end{aligned}$$

since $\frac{n}{u}\ge \frac{1}{p}$ implies $\frac{np}{u}\ge 1$ and therefore, the sum in the last line above diverges. $\square$

### Remark 2.8

(i) The special case when $p=u$ and $\mathcal {M}_{u,p}(\Omega )=L_p(\Omega )$ should be mentioned in this context. In this situation we have $\frac{n}{p}\ge \frac{1}{p}$, hence by Theorem 2.7 (ii) the three approaches differ for Lebesgue spaces (without the preceding weight factor).
(ii) Moreover, just for completeness, it would be nice to know under what conditions the different approaches always coincide or differ, but we have not pursued this idea further.
(iii) Let us finally mention, that embeddings within the scales of Morrey spaces $\mathcal {M}_{u,p}(\Omega )$, $\mathbb {M}_{u,p}(\Omega )$ or $\mathfrak {M}_{u,p}(\Omega )$, can never be compact. This simply follows by the above embeddings and the well-known fact, that embeddings between Lebesgue spaces, $L_{u_1}(\Omega ) \hookrightarrow L_{u_2}(\Omega )$ with $u_1\ge u_2$ are continuous for any bounded $\Omega \subset \mathbb {R}^n$, but never compact, cf. [25, p. 95]. For the spaces $\mathcal {M}_{u,p}(\Omega )$ this has already been observed in [12, Cor. 4.10].

### Growth Envelopes for Morrey Spaces $\mathcal {M}_{u,p}(\Omega )$

We now turn our attention towards the Morrey spaces $\mathcal {M}_{u,p}(\Omega )$, $0<p\le u<\infty$. One can easily see that

$$\begin{aligned} L_u(\Omega )\hookrightarrow \mathcal {M}_{u,p}(\Omega )\hookrightarrow L_p(\Omega ). \end{aligned}$$

In particular, the embedding on the right-hand side follows immediately from the definition. Our aim now is to tackle the question whether $L_p(\Omega )$ is indeed the best Lebesgue-type space in which the Morrey spaces can be embedded. We will study embeddings into the scale of Lorentz spaces (which can be considered as refined $L_p$ spaces) and try to obtain some optimal (sharp) results. This problem can be rephrased in terms of growth envelopes as defined by Haroske and Triebel (see [8, 31], where more details and references on the subject can be found). Therefore, we shall briefly recall the concept before we present our results. As an application of the computed growth envelopes we will obtain some answers regarding sharp embeddings and Hardy-type inequalities for Morrey spaces.

Let for some measurable $$f\in M(\Omega )$$ its decreasing rearrangement $$ f^*$$ be defined as usual,

$$\begin{aligned} f^{*}(t) = \inf \left\{ s\ge 0 : \left| \{ x\in \Omega : |f(x)|>s\}\right| \le t\right\} ,\qquad 0\le t\le |\Omega |. \end{aligned}$$

#### Definition 2.9

Let $X\subset M(\Omega )$ be some quasi-Banach function space on $\Omega$.

- (i)    Let  be defined by 2.22 The *growth envelope function* of *X* is the class  of functions $g:(0,\varepsilon ]\rightarrow [0,\infty )$, for some $\varepsilon >0$, such that  in $(0,\varepsilon ]$. For convenience, we do not distinguish between representative and equivalence class. Therefore, any representative function of the class will be called as well growth envelope function and sometimes we also denote a particular representative by .
- (ii)    Assume $X\not \hookrightarrow L_\infty (\Omega )$. Let  (or an equivalent function) be continuously differentiable. Then the number , , is defined as the infimum of all numbers *v*, $0<v\le \infty$, such that 2.23 (with the usual modification if $v=\infty$) holds for some $c>0$ and all $f\in X$. The couple  is called *(local) growth envelope* for the function space *X*.

#### Remark 2.10

Obviously, (2.23) holds for $v=\infty$ in any case, but—depending upon the underlying function space *X*—there might be some smaller $v_0$ such that (2.23) is still satisfied (and therefore also for all $v\in [v_0,\infty ]$ since the left-hand side of (2.23) is monotonically ordered in *v*), cf. [31, Prop. 12.2].

For the fundamental function $\varphi _X$ of some rearrangement invariant Banach function space $X=X(\mathbb {R}^n)$, defined by $\varphi _X(t)=\Vert \chi _{A_t}|X\Vert$, where $A_t\subset \mathbb {R}^n$ with $|A_t|=t$, it was proven in [8, Sect. 3.3] that


We recall some useful properties of growth envelopes.

#### Proposition 2.11

- (i)    Let $X_i\not \hookrightarrow L_\infty$, $i=1,2$, be some function spaces on $\Omega$. Then $X_1\hookrightarrow X_2$ implies that there is some positive constant *c* such that for all $t>0$, 2.24
- (ii)    We have $X\hookrightarrow L_\infty$ if, and only if,  is bounded.
- (iii)    Let $X_i$, $i=1,2$, be some function spaces on $\Omega$ with $X_1\hookrightarrow X_2$. Assume for their growth envelope functions  for some $\varepsilon >0$. Then we get for the corresponding indices , $i=1,2$, that

This result coincides with [8, Props. 3.4, 4.5].

#### Example 2.12

If $X=L_{p,q}(\Omega )$, $0<p<\infty$, $0<q\le \infty$, are the usual Lorentz spaces, then it is shown in [8, Thm. 4.7, Cor. 10.14] that2.25Recall that left-hand side of (2.23) with $v=q$ is an equivalent quasi-norm in $L_{p,q}(\Omega )$,

2.26 $$\begin{aligned} \left( \int _0^{|\Omega |}\left( t^{\frac{1}{p}}f^{*}(t)\right) ^q\frac{\mathrm {d}t}{t}\right) ^{\frac{1}{q}}\sim \Vert f|L_{p,q}(\Omega )\Vert . \end{aligned}$$

We now study growth envelopes of the Morrey spaces $\mathcal {M}_{u,p}(\Omega )$. The problem is delicate. On $\mathbb {R}^n$ the results from [9, Th. 3.7] establish the non-existence of growth envelopes, since it is shown there that whenever $0<p<u<\infty$, then2.27However, the situation for bounded domains is completely different. In this case we have the embeddings $L_u(\Omega )\hookrightarrow \mathcal {M}_{u,p}(\Omega )\hookrightarrow L_p(\Omega )$, which immediately give upper and lower bounds for the growth envelope function. The ideas for the theorem to come are taken from [32].

#### Theorem 2.13

Let $\Omega \subset \mathbb {R}^n$ be a bounded domain of type *A*, and let $0<p\le u<\infty$. Then2.28


#### Proof

*Step 1.*   We assume in the proof that $p<u$. The case $p=u$ is known since $\mathcal {M}_{p,p}(\Omega ) = L_p(\Omega )$. Computing the growth envelope function  the upper estimate follows immediately from the embedding $\mathcal {M}_{u,p}(\Omega )\hookrightarrow L_p(\Omega )$, which givesas desired. In order to compute the lower estimate we assume for simplicity that the domain $\Omega$ contains the unit cube $Q_{0,0}=[0,1]^n$, otherwise one can rescale the argument. Let $Q_{j,k}$, $j\in \mathbb {N}_0$ and $k\in \mathbb {Z}^n$, denote the dyadic cube by $2^{-j}k+ [0,2^{-j}]^n$. We adopt the method used in the proof of Theorem 3.1 in [11], cf. also the proof of Theorem 3.2 in [10]. For $0<\nu$ we put

$$\begin{aligned} k_\nu = \lfloor 2^{n\nu \left( 1 - \frac{p}{u}\right) } \rfloor , \end{aligned}$$

where $\lfloor x \rfloor =\max \{l\in \mathbb {Z}: l \le x\}$. Then $1\le k_\nu < 2^{n\nu }$ and there exists $c_{p, u} >0$ such that

2.29 $$\begin{aligned} k_\nu \le \ c_{p, u}\ 2^{n(\nu - \mu )} k_\mu , \qquad \text {if}\qquad 0< \mu \le \nu . \end{aligned}$$

For convenience let us assume that $c_{p, u}= 1$ (otherwise the argument below has to be modified in an obvious way). For any $j>0$ we define a finite sequence $\lambda _{j,m}$, where $m\in \{k:\; Q_{j,k}\subset Q_{0,0}\}$. The sequence takes only two values 0 and 1. Moreover the value 1 is taken $k_j$ times. It was proved in [11], cf. also [10], that the sequence can be chosen in such a way that for any $0<\nu <j$ and any cube $Q_{\nu ,k}\subset Q_{0,0}$, the subsequence $\{\lambda _{j,m}: Q_{j,m}\subset Q_{\nu ,k} \}$ contains at most $k_{j-\nu }$ elements that equal 1. We consider the functions

$$\begin{aligned} f_j(x) = 2^{\frac{nj}{u}}\sum _{m} \lambda _{j,m}\chi _{j,m}(x) . \end{aligned}$$

The function $f_j$ belongs to $\mathcal {M}_{u,p}(\Omega )$, which can be seen as follows. Since an equivalent norm in $\mathcal {M}_{u,p}(\Omega )$ can be defined by taking the supremum over dyadic cubes we see that

2.30 $$\begin{aligned} \Vert f_j|\mathcal {M}_{u,p}(\Omega )\Vert\sim & {} \sup _{\mathop {Q_{\nu ,k}\subset Q_{0,0}}\limits ^{\nu : 0\le \nu \le j} } |Q_{\nu ,k}|^{\frac{1}{u} - \frac{1}{p}} \left( \int _{Q_{\nu ,k}} |f_j(x)|^p \mathrm {d}x\right) ^{1/p} \nonumber \\\le & {} 2^{\frac{n j}{u}} \sup _{\nu : 0\le \nu \le j} 2^{-\nu n\left( \frac{1}{u} - \frac{1}{p}\right) }\left( k_{j-\nu }2^{-jn}\right) ^{\frac{1}{p}} \le C, \end{aligned}$$

and the constant *C* used in the last inequality is independent of *j*. The function $f_j$ is a simple function defined on a set of measure $2^{-jn}k_j\sim 2^{\frac{-jnp}{u}}$, which takes the value $2^{\frac{nj}{u}}$ on this set, so

2.31 $$\begin{aligned} f_j^*\left( 2^{\frac{-jnp}{u}}\right) \sim 2^{\frac{nj}{u}}=\left( 2^{\frac{-jnp}{u}}\right) ^{-1/p}\, . \end{aligned}$$

Now, the desired estimate from belowfollows from (2.30) and (2.31).

*Step 2*.   For the additional index we first deal with the lower bound. We use a refined version of the last construction. Once more we assume that the unit cube is contained in $\Omega$. Let $k_\nu = \lfloor 2^{n\nu (1-\frac{p}{u})} \rfloor$. We choose $\nu$ such that $n\nu (1-\frac{p}{u})\ge 1$. First we consider the case when $n\nu (1-\frac{p}{u})\in \mathbb {N}$, i.e. $k_\nu = 2^{n\nu (1-\frac{p}{u})}$. Furthermore, we put $\varkappa _\nu =2^{n\nu } -k_\nu$ and take the sequence $\lambda _{\nu ,\ell }$ described in the first step of the proof. The sequence takes the value 1 for $k_\nu$ cubes of size $2^{-n\nu }$ contained in $Q_{0,0}$ and the value 0 for $\varkappa _\nu$ similar cubes. Now we define by induction sequences $\lambda _{j\nu ,\ell }$ for $j>1$. First we extend the sequence $\lambda _{\nu ,\ell }$ periodically to other cubes of size 1. More precisely, if $Q_{\nu ,\ell }$ is not contained in $Q_{0,0}$, then there exists exactly one cube $Q_{\nu ,m} \subset Q_{0,0}$ such that $\ell _i\equiv m_i$ mod $2^\nu$, $i=1,\ldots , n$. We put $\lambda _{\nu ,\ell } = \lambda _{\nu ,m}$. Let now $j>1$. If $Q_{j\nu ,\ell }\subset Q_{(j-1)\nu ,m} \subset Q_{0,0}$ and $\lambda _{(j-1)\nu ,m}=0$, then $\lambda _{j\nu ,\ell }=0$. If $Q_{j\nu ,\ell }\subset Q_{(j-1)\nu ,m} \subset Q_{0,0}$ and $\lambda _{(j-1)\nu ,m}=1$, then we rescale the unit cube $Q_{0,0}$ to the cube $Q_{(j-1)\nu ,m}$. Thus $\lambda _{j\nu ,l}$ has value 0 or 1 depending on whether the sequence $\lambda _{\nu ,l}$ has value 0 or 1 on the corresponding rescaled subcube from $Q_{0,0}$.

Roughly speaking, we clone the cube $Q_{0,0}$ on any subcube where the sequence takes value 1 and repeat this construction in each step. The basic idea is illustrated below for the first step, i.e. $j=2$, with parameters $n=2$, $\nu =2$, $p=1$, $u=2$. Hence, $k_{\nu }=2^{4(1-\frac{1}{2})}=4$ and $\varkappa _{\nu }=16-4=12$. 
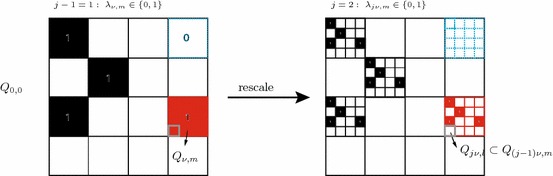


We define the function *f* supported in the cube $Q_{0,0}$ via the formula

2.32 $$\begin{aligned} f(x)= \sum _{j=1}^\infty \gamma _j \sum _m \lambda _{j\nu ,m}\chi _{j\nu ,m}(x) . \end{aligned}$$

We postpone the definition of the sequence $(\gamma _j)_j$ for a moment. The function *f* takes the value 0 on $\varkappa _\nu$ cubes of size $2^{-n\nu }$, the value $\gamma _1$ on $k_\nu \varkappa _\nu$ cubes of size $2^{-2n\nu }$ and, by induction, the value $\gamma _1+\cdots +\gamma _\ell$ on $k_\nu ^\ell \varkappa _\nu$ cubes of size $2^{-(\ell +1)\nu n}$. We choose $\lambda >0$ such that $\lambda p> 1$ and put

$$\begin{aligned} \gamma _1 =\,&2^{\nu \frac{n}{u} } ,\\ \gamma _2 =\,&2^{2\nu \frac{n}{u} }2^{-\lambda } - \gamma _1 = 2^{\nu \frac{n}{u} }\Big ( 2^{\nu \frac{n}{u} } 2^{-\lambda } -1 \Big ), \end{aligned}$$

and

$$\begin{aligned} \gamma _j = 2^{j\nu \frac{n}{u} } j^{-\lambda } - \sum _{i=1}^{j-1} \gamma _i = 2^{\nu (j-1)\frac{n}{u} } (j-1)^{-\lambda }\bigg (2^{\nu \frac{n}{u} } \Big (\frac{j-1}{j}\Big )^{\lambda } -1\bigg ) . \end{aligned}$$

The numbers $\gamma _\ell$ are positive for sufficiently large $\ell$ so the function *f* is pointwise well defined. We show that $f\in \mathcal {M}_{u,p}(\Omega )$. If $Q=Q_{0,0}$, then

$$\begin{aligned} \left( \int _Q |f(x)|^p \mathrm {d}x\right) ^{1/p}= & {} \left( \sum _{j=1}^\infty \Big (\sum _{i=1}^j \gamma _i\Big )^p 2^{-(j+1)\nu n} k_\nu ^j \varkappa _\nu \right) ^{1/p} \\= & {} \varkappa _\nu ^{\frac{1}{p}} 2^{-\nu \frac{n}{p}} \left( \sum _{j=1}^\infty \left( 2^{j\nu \frac{n}{u}}j^{-\lambda }\right) ^p 2^{-j\nu n} 2^{n\nu j\left( 1-\frac{p}{u}\right) } \right) ^{1/p} \\= & {} \varkappa _\nu ^{\frac{1}{p}} 2^{-\nu \frac{n}{p}} \left( \sum _{j=1}^\infty j^{-\lambda p} \right) ^{1/p} =C < \infty . \end{aligned}$$

If $Q=Q_{\ell \nu ,m}$ and $\lambda _{\ell \nu ,m}=1$, then analogously

2.33 $$\begin{aligned} |Q|^{\frac{1}{u} - \frac{1}{p}} \left( \int _Q |f(x)|^p \mathrm {d}x\right) ^{1/p}= & {} 2^{-n\ell \nu \left( \frac{1}{u} - \frac{1}{p}\right) } \nonumber \\&\times \left( \sum _{j=\ell -1}^\infty \Big (\sum _{i=1}^j \gamma _i\Big )^p 2^{-(j+1)\nu n} k_\nu ^{j-\ell +1}\varkappa _\nu \right) ^{1/p} \nonumber \\= & {} \varkappa _\nu ^{\frac{1}{p}} 2^{-\nu \frac{n}{u}} 2^{-n\ell \nu \left( \frac{1}{u} - \frac{1}{p}\right) } \nonumber \\&\times \left( \sum _{j=\ell -1}^\infty \left( 2^{j\nu \frac{n}{u}}j^{-\lambda }\right) ^p 2^{-j\nu n} 2^{n\nu (j-\ell )\left( 1-\frac{p}{u}\right) } \right) ^{1/p} \nonumber \\= & {} \left( \sum _{j=\ell -1}^\infty j^{-\lambda p} \right) ^{1/p} \le C<\infty . \end{aligned}$$

If $Q=Q_{\ell \nu ,m}$ and $\lambda _{\ell \nu ,m}=0$, then the sum $\left( \sum _{j=\ell -1}^\infty ...\right) ^{1/p}$ in (2.33) is reduced to $\left( \Big (\sum _{i=1}^{\ell -1} \gamma _i\Big )^p 2^{-\nu \ell n}\varkappa _\nu \right) ^{1/p}$. So in this case the corresponding integral is smaller.

If *Q* is any dyadic cube in $Q_{0,0}$, then one can find $\ell \in \mathbb {N}$ such that $Q_{\ell \nu , m} \subset Q\subset Q_{(\ell -1)\nu , k}$ for some $m,k \in \mathbb {Z}^n$. So it follows from (2.33) that

2.34 $$\begin{aligned} |Q|^{\frac{1}{u} - \frac{1}{p}} \left( \int _Q |f(x)|^p \mathrm {d}x\right) ^{1/p} \le C_{\nu }\, <\, \infty . \end{aligned}$$

For the rearrangement $f^{*}$ let first $t= k_\nu ^j 2^{-(j+1)n\nu }\varkappa _\nu = 2^{-n\nu j \frac{p}{u}} \varkappa _\nu 2^{-\nu n}$. Then by the construction

2.35 $$\begin{aligned} f^*\left( 2^{-n\nu j \frac{p}{u}} \varkappa _\nu 2^{-\nu n} \right) = \sum _{i=1}^j\gamma _j=2^{nj\nu \frac{1}{u}} j^{-\lambda } , \end{aligned}$$

hence,

$$\begin{aligned} f^{*}(t)\sim t^{-\frac{1}{p}}|\log t|^{-\lambda }, \qquad 0< t \le \varepsilon . \end{aligned}$$

Let $v<p$ and choose $\lambda$ such that $v\lambda =1< \lambda p$. Then one hasThis proves  for *u* and $p<u$ such that $n\nu (1-\frac{p}{u})\in \mathbb {N}$ for some $\nu \in \mathbb {N}$. In the general case when $n\nu (1-\frac{p}{u})\notin \mathbb {N}$ we can always find $\nu \in \mathbb {N}$ such that $p< u < p\nu$ . If $u_1=p\nu$, then $n\nu (1-\frac{p}{u_1})=n\nu (1-\frac{1}{\nu })=n\nu -n\in \mathbb {N}$,Therefore Proposition 2.11 gives . The converse follows fromwhich yields . $\square$

#### Remark 2.14

We would like to draw the reader’s attention again to the big change when replacing $\Omega$ by $\mathbb {R}^n$ in the definition of Morrey spaces $\mathcal {M}_{u,p}$. As recalled in (2.27) we proved in [9] that whenever $p<u$, then the growth envelope function is infinite for any $t>0$, whereas now, for bounded $\Omega$, we always have , $t>0$.

We point out that the $\mathrm {bmo}$-spaces show a similar change in behaviour in terms of growth envelopes as the Morrey spaces $\mathcal {M}_{u,p}$ that are studied here. The inhomogeneous space $\mathrm {bmo}(\mathbb {R}^n)$ consists of those locally integrable functions with bounded mean oscillation for which

$$\begin{aligned} \Vert f|\mathrm {bmo}(\mathbb {R}^n)\Vert =\sup _{|Q|\le 1}\frac{1}{|Q|}\int _Q|f(x)-f_Q|\mathrm {d}x+\sup _{|Q|>1}\frac{1}{|Q|}\int _Q|f(x)|\mathrm {d}x<\infty , \end{aligned}$$

where *Q* denotes cubes in $\mathbb {R}^n$ and $f_Q$ is the mean value of *f* with respect to *Q*. Furthermore, $\mathrm {bmo}(\Omega )$ is defined by restriction of $\mathrm {bmo}(\mathbb {R}^n)$ on $\Omega$. For these spaces it is shown in [31, Sect. 13.7] thatwhereas on bounded domains $\Omega$ one has


#### Theorem 2.15

Let $\Omega \subset \mathbb {R}^n$ be a bounded Lipschitz domain, and let $0<p\le u<\infty$. Then2.36


#### Proof

The first line in (2.36) is simply a consequence of Theorems 2.7 (i) and 2.13. So it remains to deal with the case $\frac{n}{u}>\frac{1}{p}$. Obviously, the upper estimate for  directly follows from the embedding (2.11) in Theorem 2.5 (ii) in view of (2.25) in Example 2.12 and (2.24) in Proposition 2.11 (i). Now we verify that  as $t\rightarrow 0$. We consider the functions

$$\begin{aligned} g_J=2^{\frac{Jn}{u}}\chi _{S_J}, \qquad J\in \mathbb {N}_0. \end{aligned}$$

Then

$$\begin{aligned} \Vert g_J|\mathfrak {M}_{u,p}(\Omega )\Vert= & {} \sup _{x\in \Omega , j\ge j_x}2^{-jn\left( \frac{1}{u}-\frac{1}{p}\right) }\left[ \int _{B(x,2^{-j})}|g_J(y)|^p\mathrm {d}y\right] ^{\frac{1}{p}}\\\lesssim & {} \sup _{j\ge J} 2^{-jn\left( \frac{1}{u}-\frac{1}{p}\right) }2^{\frac{Jn}{u}} 2^{-j\frac{n}{p}}= \sup _{j\ge J} 2^{-\frac{n}{u}(j-J)}\lesssim 1. \end{aligned}$$

Furthermore, since $|S_J|\sim 2^{-J}$, we have

$$\begin{aligned} g_J^{*}(t)\sim 2^{\frac{Jn}{u}}, \qquad t\sim 2^{-J},\quad J\in \mathbb {N}_0, \end{aligned}$$

which gives  as $t\rightarrow 0$.

We deal with the index in the second case, i.e. when $\frac{n}{u}<\frac{1}{p}$. Plainly we only need to disprove that . Let

$$\begin{aligned} g=\sum _{J=0}^{\infty } g_J, \qquad g_J=2^{\frac{Jn}{u}}\chi _{S_J}. \end{aligned}$$

For the norm of *g* it follows by the above arguments and the construction that $\Vert g|\mathfrak {M}_{u,p}(\Omega )\Vert \lesssim 1$. We now calculate the rearrangement $g^{*}$. Let first $s=2^k$, $k\in \mathbb {N}$. Then we see that

$$\begin{aligned} |g(x)|>s\iff & {} \exists \ J: \ x\in S_J, \ 2^{\frac{Jn}{u}}>2^k\quad \iff \quad \exists \ J\ge J_k: \ x\in S_J, \end{aligned}$$

with $$J_k = \lfloor k\frac{u}{n} \rfloor +1$$. Thus

$$\begin{aligned} \{x: \ |g(x)|>2^k\} \sim \sum _{J=J_k}^{\infty }|S_J|\sim 2^{-J_k}\sim 2^{-k\frac{u}{n}} \end{aligned}$$

and $\{x: |g(x)|>s\}\sim s^{-\frac{u}{n}}$. From this we obtain

$$\begin{aligned} g^{*}(t)= \inf \left\{ s>0: \ s^{-\frac{u}{n}}\le t\right\} \ =\ \inf \left\{ s>0: \ s\ge t^{-\frac{n}{u}}\right\} \sim t^{-\frac{n}{u}}, \qquad 0<t\le \varepsilon . \end{aligned}$$

But then for arbitrary $v<\infty$,which yields  and concludes the proof. $\square$

#### Remark 2.16

(i) In the unpublished notes [32, Cor. 2.16] the case $\frac{n}{u}>\frac{1}{p}$ is obtained. This was in fact our starting point for the study of growth envelopes in Morrey spaces on domains. For the spaces $\mathfrak {M}^*_{u,p}(\Omega )$ introduced in (2.19), recall Remark 2.6, Triebel proved in [32] in a similar way that 2.37 Comparing this result with (2.36), second line, one observes that the growth envelope functions for $\mathfrak {M}_{u,p}(\Omega )$ and $\mathfrak {M}^*_{u,p}(\Omega )$ are the same, whereas the corresponding indices differ. This well reflects the different constructions in (2.4) and (2.19) based on the (outer) $\ell _\infty$- or $\ell _p$-norm, respectively.
(ii) One of the most interesting questions now is surely the gap $\frac{n}{u}=\frac{1}{p}$ in Theorem 2.15. Though some of our arguments still work and provide upper and lower bounds, a complete answer is missing yet. At the moment we do not even have a well-founded guess in this case.
(iii) By Theorems 2.7 (i) and 2.13 we immediately get 2.38

**Applications: Optimal embeddings and Hardy-type inequalities** Observe that (2.25) and (2.28) imply for $0<p\le u<\infty$,We already know that $\mathcal {M}_{u,p}(\Omega )\hookrightarrow L_p(\Omega )$, whereas their growth envelopes even coincide. This can be interpreted as $L_{p}(\Omega )$ being indeed the best possible space within the Lorentz (Lebesgue) scale in which $\mathcal {M}_{u,p}(\Omega )$ can be embedded continuously. On the other hand this can also be understood in the sense that $L_p(\Omega )$ is as good as $\mathcal {M}_{u,p}(\Omega )$, as far as only the growth of the unbounded functions belonging to the spaces under consideration is concerned.

As mentioned several times already, this situation is completely different on $\mathbb {R}^n$. In [9, Th. 3.7] it was shown that for $\mathcal {M}_{u,p}(\mathbb {R}^n)$ the growth envelope function does not exist, since


#### Remark 2.17

Having in mind what was said above, the growth envelope resulting from [32] show that concerning optimal embeddings, the situation is quite different for the spaces $\mathfrak {M}_{u,p}(\Omega )$. By (2.36) it follows that

$$\begin{aligned} \mathfrak {M}_{u,p}(\Omega )\hookrightarrow L_{\frac{u}{n},\infty }(\Omega ), \qquad \text {if}\quad \frac{n}{u}>\frac{1}{p}, \end{aligned}$$

is the best possible embedding into the scale of Lorentz spaces.

Finally, we state what can be said about Hardy-type inequalities for Morrey spaces. This follows immediately from our above results together with the properties of $f^*$ and the fact that, given $\varkappa$ non-negative on $(0,\varepsilon ]$,holds for some $c>0$ and all $f\in X$, $\Vert f|X\Vert \le 1$, if, and only if, $$ \ \varkappa \ $$ is bounded, cf. [8, Prop. 3.4(v)].

#### Corollary 2.18

Let $\Omega$ be a bounded domain of type *A*, $0<p\le u<\infty$, $\varepsilon >0$ small, and $\varkappa (t)$ be a positive monotonically decreasing function on $(0,\varepsilon ]$, and let $0<v\le \infty$. Then

2.39 $$\begin{aligned} \Big (\int _0^{\varepsilon }\left[ \varkappa (t)t^{\frac{1}{p}}f^{*}(t)\right] ^{v}\frac{\mathrm {d}t}{t}\Big )^{\frac{1}{v}} \le c \Vert f|\mathcal {M}_{u,p}(\Omega )\Vert , \end{aligned}$$

for some $c>0$ and all $f\in \mathcal {M}_{u,p}(\Omega )$, if, and only if, $\varkappa$ is bounded and $p\le v\le \infty$, with the usual modification

2.40 $$\begin{aligned} \sup _{t\in (0,\varepsilon )}\varkappa (t)t^{\frac{1}{p}}f^{*}(t)\le c \Vert f|\mathcal {M}_{u,p}(\Omega )\Vert , \end{aligned}$$

if $v=\infty$. In particular, if $\varkappa$ is an arbitrary non-negative function on $(0,\varepsilon ]$, then (2.40) holds if, and only if, $\varkappa$ is bounded.

#### Proof

This is an immediate consequence of . $\square$

For the Morrey spaces $\mathfrak {M}_{u,p}(\Omega )$ the analogue reads as follows.

#### Corollary 2.19

Let $\Omega$ be a bounded domain of type *A*, $0<p\le u<\infty$, $\varepsilon >0$ small, and $\varkappa (t)$ be a positive monotonically decreasing function on $(0,\varepsilon ]$, and let $0<v\le \infty$. Then

2.41 $$\begin{aligned} \Big (\int _0^{\varepsilon }\left[ \varkappa (t)t^{-a}f^{*}(t)\right] ^{v}\frac{\mathrm {d}t}{t}\Big )^{\frac{1}{v}} \le c \Vert f|\mathfrak {M}_{u,p}(\Omega )\Vert , \end{aligned}$$

for some $c>0$ and all $f\in \mathfrak {M}_{u,p}(\Omega )$, if, and only if, $\varkappa$ is bounded and

$$\begin{aligned} {\left\{ \begin{array}{ll} a=\frac{1}{p}\quad \text {and}\quad p\le v\le \infty , &{} \text {if}\quad \frac{n}{u}<\frac{1}{p},\\ a=\frac{n}{u}\quad \text {and}\quad v=\infty , &{} \text {if}\quad \frac{n}{u}>\frac{1}{p}, \end{array}\right. } \end{aligned}$$

with the usual modification

2.42 $$\begin{aligned} \sup _{t\in (0,\varepsilon )}\varkappa (t)t^{-a}f^{*}(t)\le c \Vert f|\mathfrak {M}_{u,p}(\Omega )\Vert , \end{aligned}$$

if $v=\infty$. In particular, if $\varkappa$ is an arbitrary non-negative function on $(0,\varepsilon ]$, then (2.42) holds if, and only if, $\varkappa$ is bounded.

#### Proof

This is an immediate consequence of the growth envelope results in Theorem 2.15. $\square$

#### Remark 2.20

Plainly one can also formulate counterparts of the above corollaries for spaces $\mathfrak {M}^*_{u,p}(\Omega )$, $\frac{n}{u}>\frac{1}{p}$, and $\mathbb {M}_{u,p}(\Omega )$, $\frac{n}{u}<\frac{1}{p}$, in view of (2.37) and (2.38), respectively. This is left to the reader.
